# miR172-Mediated Repression of *APETALA2*-like Genes Regulates Floral Meristem Activity During Double-Flower Formation in *Camellia japonica*

**DOI:** 10.3390/ijms27062769

**Published:** 2026-03-18

**Authors:** Lusi Huang, Yifan Yu, Yixuan Luo, Yi Feng, Xiaoping Wang, Hengfu Yin

**Affiliations:** 1 State Key Laboratory of Tree Genetics and Breeding, Key Laboratory of Forest Genetics and Breeding, Research Institute of Subtropical Forestry, Chinese Academy of Forestry, Hangzhou 311400, China; 15116299041@163.com (L.H.); yyf95896065@163.com (Y.Y.); lois_luoyixuan@163.com (Y.L.); fy11071107@163.com (Y.F.); 2 College of Information Science and Technology, Nanjing Forestry University, Nanjing 210037, China; 3 Research Institute of Fast-Growing Tress, Chinese Academy of Forestry, Zhanjiang 524022, China; 4 Zhejiang Key Laboratory of Forest Genetics and Breeding, Research Institute of Subtropical Forestry, Chinese Academy of Forestry, Hangzhou 311400, China; 5 Laboratory of Plant Molecular Genetics & Crop Gene Editing, School of Life Sciences, Linyi University, Linyi 276000, China

**Keywords:** miRNA172, *APETALA2*-like, floral development, double flower, *Camellia japonica*

## Abstract

The miRNA172–*APETALA2* (*AP2*) regulatory module is a conserved mechanism governing floral development in plants. Disruption of the miR172 target sites in *AP2* genes has been shown to be key to the domestication of double flowers in ornamental species. *Camellia japonica*, a woody ornamental plant with diverse floral forms, serves as an important model for studying double-flower formation. In this study, we characterized two *AP2*-like transcription factors, *CjAP2-1* and *CjAP2-2*, which possess evolutionarily conserved miR172-binding sites and exhibit broad expression across floral tissues. To investigate the role of the miR172–*AP2* module in *C. japonica*, we identified four members of the miR172 family and demonstrated that miR172 is directly involved in the cleavage of *CjAP2-1* and *CjAP2-2* transcripts. Through bulked amplicon sequencing of cultivars with diverse floral forms, we uncovered natural variations at the miR172-binding sites of *CjAP2-1* and *CjAP2-2*, which can potentially disrupt miR172-mediated mRNA cleavage. We showed that two dinucleotide mutations (*CjAP2-1-mut5* and *CjAP2-1-mut9*) significantly reduced the miR172-mediated repression of *CjAP2-1* transcripts. Functional analysis in *Arabidopsis* revealed that overexpression of the *CjAP2-1-mut5* variant caused significant floral abnormalities, including ectopic formation of reproductive organs, loss of floral determinacy, and fusion of floral organs. Further analysis of downstream genes indicated that key regulators of floral homeotic and meristem activity were markedly altered in the transgenic plants. Our findings demonstrate that perturbations in the miR172–*AP2* regulatory relationship underlie the formation of double flowers in *C. japonica* by altering floral meristem determinacy and organ identity.

## 1. Introduction

Floral development is governed by the floral meristem, a highly organized and dynamic tissue whose cells differentiate into the distinct organ systems of the flower [1,2]. Extensive genetic and molecular studies in *Arabidopsis thaliana* and other plant species have established a regulatory framework controlling floral meristem identity and floral organ specification [3,4,5,6]. These efforts culminated in the formulation of the ABCDE model of flower development [6,7,8]. According to this model, floral organ identity is determined by the combinatorial activities of five classes of homeotic genes—A, B, C, D, and E—acting in a spatially defined manner across the concentric whorls of the floral meristem. Specifically, A-class genes (*APETALA1* [*AP1*] and *AP2*) specify sepal identity in the outermost whorl. The combined action of A- and B-class genes (*PISTILLATA* [*PI*] and *AP3*) determines petal formation in the second whorl. In the third whorl, B-class genes together with the C-class gene *AGAMOUS* (*AG*) specify stamen identity, whereas the C-class gene alone confers carpel identity in the innermost whorl. D-class genes, including *SEEDSTICK* (*STK*), *SHATTERPROOF1* (*SHP1*), and *SHP2*, act in concert with E-class proteins to specify ovule identity. The E-class genes, represented by the *SEPALLATA* (*SEP*) family, are indispensable for the identity of all floral organs, functioning as central components of higher-order transcriptional complexes [3,4,5,6,7,8].

The precise temporal and spatial expression patterns of A-, B-, C-, D-, and E-class genes are regulated by a multilayered regulatory network involving transcription factors, chromatin remodeling factors, and microRNAs (miRNAs) [9,10]. Among these, miRNAs play a crucial role in post-transcriptional gene regulation and are key determinants of floral development [11,12]. Numerous miRNA–target modules have been shown to modulate floral organ identity, patterning, and differentiation. For instance, the miR390 family regulates floral transition through the tasiRNA–*AUXIN RESPONSE FACTOR* (*ARF*) pathway [13,14,15], while miR824 targets *AG-LIKE16* (*AGL16*) to influence flowering time in *Arabidopsis* [16]. The miR169–*NUCLEAR TRANSCRIPTION FACTOR Y SUBUNIT ALPHA* (*NF-YA*) module restricts the expression domain of C-function genes in *Antirrhinum* and *Petunia* [17]. Additionally, miR156–*SQUAMOSA PROMOTER-BINDING PROTEIN-LIKE* (*SPL*) modules are required for gametophyte cell differentiation during stamen development, whereas miR319–*TEOSINTE BRANCHED1-CYCLOIDEA-PROLIFERATING CELL FACTOR* (*TCP*) and miR167–*ARF* modules contribute to stamen and pistil development, respectively [18,19,20,21]. The miR164–*CUP-SHAPED COTYLEDON/NO APICAL MERISTEM* (*CUC/NAM*) pathway regulates carpel fusion and floral organ boundary formation [22,23,24,25,26], while the miR160–*ARF* and miR165/166–*PHABULOSA* (*PHB*) pathways play pivotal roles in embryogenesis and ovule development [27,28,29]. Moreover, miR159–*V-MYB AVIAN MYELOBLASTOSIS VIRAL ONCOGENE HOMOLOG* (*MYB*), miR396–*GROWTH-REGULATING FACTOR* (*GRF*), and miR393–*TRANSPORT INHIBITOR RESPONSE 1/AUXIN SIGNALING F-BOX 2* (*TIR1/AFB2*) modules participate in multiple aspects of floral organ development by modulating transcriptional regulators and hormone signaling pathways [30,31,32,33]. Collectively, these studies underscore that floral organ formation is orchestrated by a complex and interconnected regulatory network comprising multiple miRNA-mediated pathways.

The miR172-*AP2* regulatory module is a conserved molecular mechanism that critically governs floral organ identity and flowering time in angiosperms. In *Arabidopsis*, miR172 post-transcriptionally represses *AP2* and several *AP2*-like genes—including *TARGET OF EAT1* (*TOE1*), *TOE2*, *TOE3*, *SCHLAFMÜTZE* (*SMZ*), and *SCHNARCHZAPFEN* (*SNZ*)—all of which function as key developmental regulators [34,35,36,37]. Overexpression of miR172 members leads to premature flowering and homeotic transformations of floral organs, similar to the phenotypes of mutants in miR172-resistant forms of these *AP2*-like genes [35,38]. Notably, disruptions in the miR172-*AP2* interaction—particularly through mutations in the miR172-binding site of *AP2*-like transcripts—have been repeatedly shown to cause double-flower phenotypes across diverse ornamental species. For instance, in peach (*Prunus persica*), mei (*Prunus mume*), and rose (*Rosa* spp.), such mutations are found to impair miR172-mediated cleavage, resulting in ectopic or prolonged expression of *AP2*-like genes and the consequent conversion of stamens and carpels into petaloid organs, thereby increasing petal number [39,40,41,42]. In carnation (*Dianthus caryophyllus*), similar disruptions—either through single-nucleotide polymorphisms (SNPs) or transposable element insertions in the miR172 target site of *AP2*-like genes—prevent miRNA binding and cleavage, resulting in the development of double flowers [43,44]. Together, these findings underscore the central role of the miR172-*AP2* module in the molecular control of floral architecture and its recurrent recruitment in the domestication and diversification of ornamental flowers.

*Camellia japonica* is a widely cultivated ornamental species with substantial horticultural and economic value. Double-flowered cultivars of *C. japonica* exhibit striking alterations in floral organ identity, morphology, and number [45,46]. A comparative transcriptomic analysis has identified the homologs of floral homeotic genes (A-, B-, C- and E-class genes) in *C. japonica* [47]. In particular, the changes in the expression patterns of A- and C-class genes are closely associated with double-flower formation in *C. japonica* [45,46]. In *Camellia azalea*, integrated small RNA and transcriptome sequencing approaches have proven effective in identifying key regulatory genes, including both conserved and novel miRNAs, in floral organs [12]. In addition, an ovule-specific miRNA, miR5179, targets a B-class gene *DEFICIENS* (*DEF*) in *C. japonica* [48]. However, whether the miR172–*AP2* regulatory module participates in the control of floral development in *C. japonica* remains largely unexplored.

In the present study, two homologs of the *AP2*-like transcription factor family (*CjAP2-1* and *CjAP2-2*) are identified from *C. japonica*, which are detectable across all floral organs but expressed at the highest level in carpels. Consistently, the precursors of miR172 are highly expressed in stamens and carpels, suggesting that miR172 may have a degrading effect on the mRNA of *CjAP2*. Notably, natural variations are observed at the miR172 target site, and the two dinucleotide substitutions in *CjAP2-1* are expected to disrupt miR172-mediated mRNA cleavage. Furthermore, functional analysis in *Arabidopsis* demonstrates that the miR172–*CjAP2* regulatory module plays a critical role in modulating floral organ formation. 

## 2. Results

### 2.1. Identification of CjAP2 Transcription Factors and Expression Profiling in Different Floral Types

**Figure 1:**
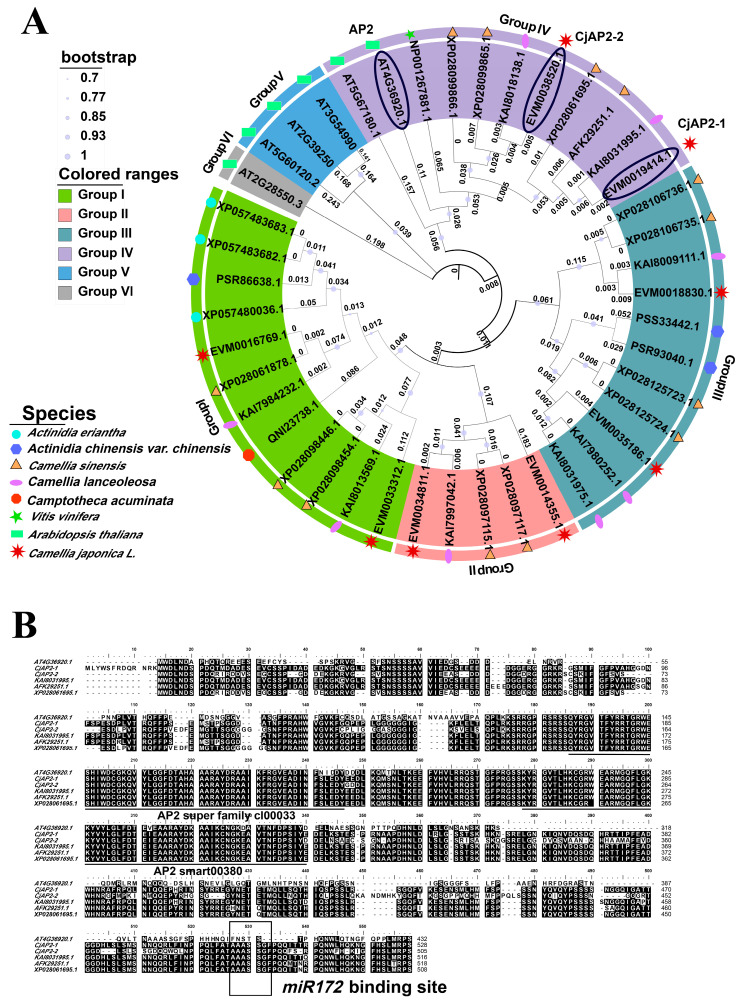
Phylogenetic relationships and expression patterns of *CjAP2* genes in *C. japonica*. (**A**) Neighbor-joining (NJ) phylogenetic tree of AP2–TOE family proteins from *Camellia* species and representative eudicots. Sequences from species other than *C. japonica* were retrieved from the GenBank database. Node shapes indicate the species of origin and the number represents the bootstrap value. The AP2 circled is the query sequence to perform a BLAST search. *CjAP2-1* and *CjAP2-2* circled are research objects in this study. (**B**) Alignment of the amino acid sequences of CjAP2–TOE candidates from *Arabidopsis* and *Camellia*. The conserved AP2 domains are highlighted by lines. The putative miR172 target sites are highlighted by the box.

In order to identify the *AP2* homologous genes in *C. japonica*, we used a homology-based search strategy and the *Arabidopsis* AP2 protein sequence as the query sequence to perform a BLAST (E-value cutoff 1 × 10^−15^) search on the *C. japonica* genome database ([NCBI Bioproject accession No. PRJNA901631]) [49]. In total, eight candidate *AP2-TOE*-like genes were revealed (Figure 1A). To elucidate the evolutionary relationships among AP2 family members across eudicots, a phylogenetic tree was constructed using AP2-TOE protein sequences from multiple representative eudicot species (Figure 1A). Phylogenetic analysis indicated that two candidates from *C. japonica* clustered most closely with *Arabidopsis* AP2-TOE group members (including *AT4G36920.1* [*AP2*], *AT2G28550.1* [*TOE1*], *AT5G60120.1* [*TOE2*], etc.), which were then designated *CjAP2-1* (EVM0019414) and *CjAP2-2* (EVM0038520) (Figure 1A). According to a previous report [49], the *C. japonica* genome experienced a genome-wide duplication event, which may have resulted in multiple homologous genes and could explain why two *CjAP2* homologous genes were identified in this study. Multiple sequence alignment confirmed that both proteins contained the conserved AP2/ethylene-responsive factor (ERF) DNA-binding domain, with strong conservation of key residues characteristic of the AP2-TOE subfamily (Figure 1B). Furthermore, both genes harbored the canonical miR172 target sites, which are conserved across AP2-TOE family members (Figure 1B), indicating functional conservation within this regulatory gene group.

**Figure 2:**
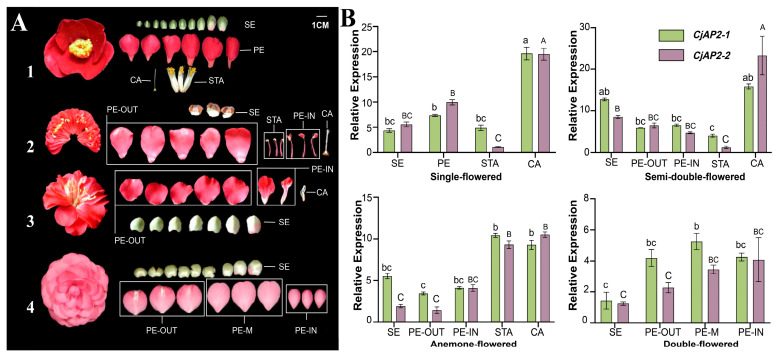
The expression profiles of *CjAP2-1* and *CjAP2-2* in floral organs. (**A**) Floral morphologies of four representative *Camellia* flower types and their corresponding structural dissections of floral organs: (1) single-flowered; (2) anemone-flowered; (3) semi-double-flowered; (4) double-flowered. (**B**) Organ-specific expression patterns of *CjAP2* genes in *C. japonica* flowers. SE, sepal; PE, petal; STA, stamen; CA, carpel; PE-OUT, outer petal; PE-M, middle petal; PE-IN, inner petal. Different uppercase letters indicate extremely significant differences (*p* < 0.01), different lowercase letters indicate significant differences (*p* < 0.05), and shared letters (e.g., AB/ab) indicate no significant difference among groups. Data represent mean ± SD (*n* = 3). Statistical significance was determined using Tukey’s HSD test.

We further examined the expression patterns of *CjAP2-1* and *CjAP2-2* across four distinct floral morphologies in *Camellia*: single-flowered, semi-double-flowered, anemone-flowered, and double-flowered forms (Figure 2A). In anemone-flowered types, the five outermost petals adjacent to the sepals were designated as normal outer petals, while the inner whorl of petaloid organs was interpreted as intermediate petaloid structures likely derived from stamens via homeotic transformation (Figure 2A). Quantitative real-time PCR (qRT-PCR) analysis showed that both *CjAP2-1* and *CjAP2-2* were expressed in all floral organs across all four flower types, albeit with subtle changes in expression patterns in different floral organs (Figure 2B). In single and semi-double flowers, the highest transcript levels of both genes were detected in carpels (Figure 2B). Notably, in anemone-flowered flowers, *CjAP2-1* and *CjAP2-2* exhibited markedly elevated expression in stamens and carpels, suggesting functional distinction associated with the types of floral organs (Figure 2B).

### 2.2. Subcellular Localization of CjAP2-1 and CjAP2-2 Proteins

**Figure 3:**
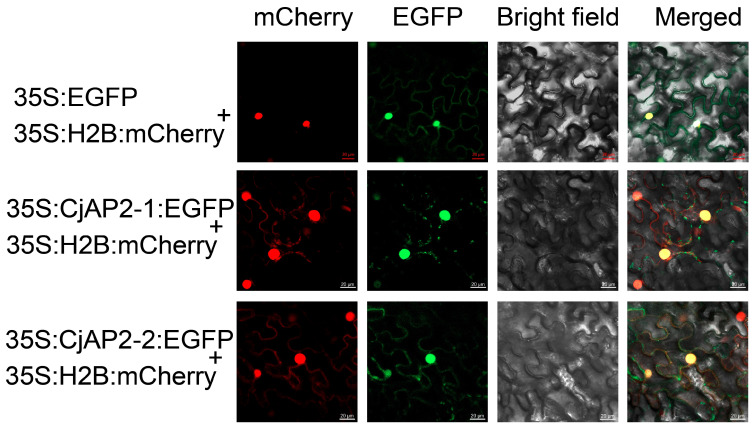
Subcellular localization of CjAP2-1 and CjAP2-2 proteins. The green fluorescence signal from the empty 35S:EGFP vector, along with the 35S:H2B nuclear marker (red fluorescence signal), is expressed in tobacco cells. The nuclear localization of the CjAP2-1–EGFP and CjAP2-2–EGFP fusion proteins was verified by the co-localization of green and red fluorescence signals.

To determine the subcellular localization of CjAP2 proteins, *CjAP2-1–EGFP* and *CjAP2-2–EGFP* fusion constructs were transiently expressed in *Nicotiana benthamiana* leaves via *Agrobacterium tumefaciens*-mediated transformation. Confocal microscopy revealed that the EGFP signals from both fusion proteins co-localized with the nuclear marker 35S:H2B:mCherry, indicating that *CjAP2-1* and *CjAP2-2* are predominantly localized in the nucleus (Figure 3). It should be pointed out that this result is the positioning information in the heterogeneous system.

### 2.3. Expression Patterns of miR172 Family Members in C. japonica

**Figure 4:**
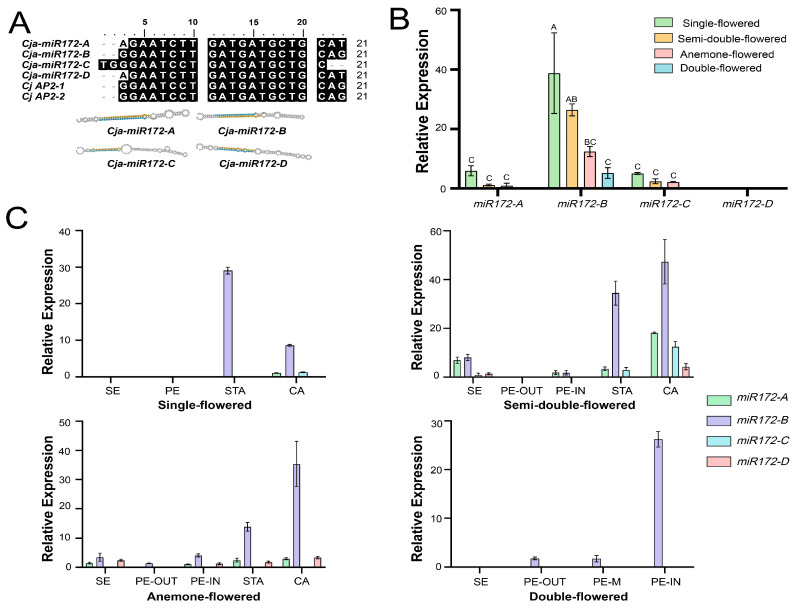
Expression characteristics of *miR172* in *C. japonica*. (**A**) Predicted secondary structure of the miR172 precursors and sequence alignment between miR172 members and their putative target sites from *CjAP2-1* and *CjAP2-2*. (**B**) Expression patterns of the miR172 precursors in floral buds of different floral types of *C. japonica*. (**C**) Relative expression levels of four miR172 members in different floral tissues of *Camellia* flower types. SE, sepal; PE, petal; STA, stamen; CA, carpel; PE-OUT, outer petal; PE-M, middle petal; PE-IN, inner petal. In B–C, different letters indicate significant differences (*p* < 0.01); shared or combined letters (e.g., AB, BC) indicate no highly significant difference. Data represent mean ± SD (*n* = 3). Tukey’s HSD test was used for multiple comparisons.

The presence of miR172 target sites suggests the function of *CjAP2-1* and *CjAP2-2* is regulated at the post-transcription level. To investigate the miR172–AP2–TOE regulatory relationship, we analyzed small RNA sequencing data from floral buds of *C. japonica* and searched for miR172 genes [47]. Four members of the miR172 family, including their stem-loop and mature sequences, were identified in *C. japonica*, designated as miR172-A, miR172-B, miR172-C, and miR172-D. Secondary structure prediction showed that all four miR172 precursors form characteristic stem-loop structures required for miRNA biogenesis (Figure 4A). Alignment of their mature sequences with the predicted miR172 target site in *CjAP2* revealed that miR172-B exhibited the highest sequence complementarity, differing by only a single nucleotide (Figure 4A).

We evaluated the expression patterns of *miR172* genes using stem-loop-specific primers. Our results showed that, among the four floral types analyzed, *miR172-B* was expressed at significantly higher levels compared to the other members of the miR172 family (Figure 4B). Further tissue-specific analysis revealed that *miR172-B* exhibited consistent high expression across the inner floral organs in all flower types, with particularly strong accumulation observed in stamens and carpels (Figure 4C). The expression of *miR172-B* was also found to be abundant in the inner petals of fully doubled flowers (Figure 4C).

### 2.4. Detection of Allelic Variation in CjAP2-1 and CjAP2-2 Genes Using Bulked Amplicon Sequencing in Camellia Cultivars

**Figure 5:**
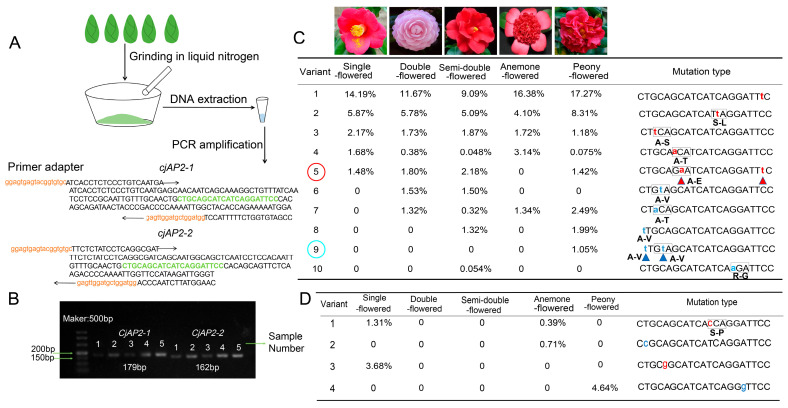
Identification of sequence variations at the miR172 target site of *CjAP2-1* and *CjAP2-2* genes using bulked amplicon sequencing. (**A**) Schematic representation of the experimental design, including DNA preparation and primer design flanking the miR172 target site. Target fragments were amplified from genomic DNA of bulked samples and analyzed using amplicon sequencing. (**B**) Electrophoretic analysis of amplified fragments from pooled samples: 1, single-flowered; 2, double-flowered; 3, semi-double-flowered; 4, anemone-flowered; 5, peony-flowered. (**C**) Frequency of sequence variation at the miR172 target site of *CjAP2-1* among different flower types. (**D**) Frequency of sequence variation at the miR172 target site of *CjAP2-2* among different flower types. In C–D, the lowercase letters indicate mutated bases. Red denotes mutation types present in both single- and double-flowered *Camellia*, whereas blue denotes mutations detected exclusively in double-flowered accessions. The red rectangles indicate the codons, and changes in amino acids are indicated by single-letter symbols under the rectangles.

The miR172-*AP2* regulation has been shown to be central to double-flower formation in several organs [39,40,41,42]. To identify sequence variations at the miR172 target site, we developed a pipeline for the efficient detection of sequence variations. First, specific primers flanking the target regions of *CjAP2-1* and *CjAP2-2* were designed, and the corresponding genomic fragments were amplified from bulked Camellia accessions (Supplementary Table S1; Figure 5A). The resulting amplicons were verified by gel electrophoresis, purified, and subsequently subjected to amplicon sequencing (Figure 5B). The sequencing results were aligned to the reference genome, and the variations at the miR172 target sites of *CjAP2-1* and *CjAP2-2* were evaluated. 

In *CjAP2-1*, a total of ten distinct mutations were identified at the miR172 target site, including eight single-nucleotide substitutions and two dinucleotide mutations, designated *CjAP2-1-mut5* and *CjAP2-1-mut9*. *CjAP2-1-mut5* was detected in both single- and double-flowered accessions, whereas *CjAP2-1-mut9* was exclusively associated with double-flowered *Camellia* (Figure 5C). In contrast, only four single-nucleotide mutations were detected at the miR172 target site of *CjAP2-2*, and only single-nucleotide substitutions were identified (Figure 5D). The translation analysis of the sequence variation in the miR172 target site region of the *CjAP2-1* and *CjAP2-2* genes showed that no premature stop codon was introduced.

### 2.5. Mutations at the miR172 Target Sites Attenuate miRNA-Mediated Cleavage of CjAP2-1 Transcripts

**Figure 6:**
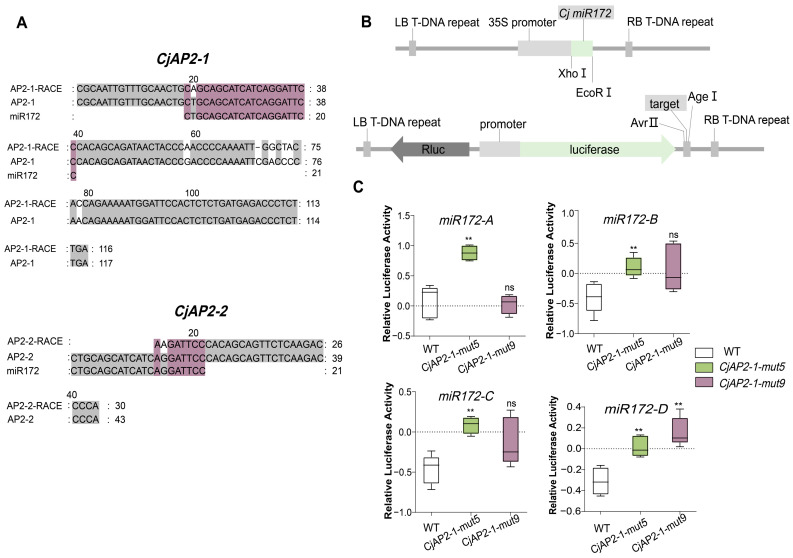
Differential disruption of miR172-mediated repression by target-site mutations in *CjAP2-1* and *CjAP2-2*. (**A**) The verification of miR172-directed cleavage of *CjAP2-1* and *CjAP2-2* transcripts by 5′-RACE analysis. Comparison of truncated *CjAP2* mRNA sequences with the corresponding miR172 target sequences. Numbers represent the number of nucleotides. (**B**) Schematic diagram of the dual-luciferase reporter system, including the pGreen_GUS_competitor and pGreen_dualluc_3′UTR_sensor vectors. Rluc indicates Renilla luciferase, and LUC indicates firefly luciferase. (**C**) Effects of four miRNAs on the luciferase activity of *CjAP2-1-mut5* and *CjAP2-1-mut9*, assessed using the dual-luciferase assay. The unmutated *CjAP2-1* construct was used as the wild-type (WT) control. Five biological replicates are used for each analysis. ns, not significant; ** *p*-value < 0.01.

To confirm miR172-directed cleavage of *CjAP2-1* and *CjAP2-2* transcripts, 5′-RACE analysis was performed using carpel tissue from the wild *C. japonica* ‘Naidong’. Cleavage products corresponding to *CjAP2-1* and *CjAP2-2* were detected at positions proximal to the predicted miR172 target site, confirming direct miRNA-mediated cleavage in vivo (Figure 6A). It should be noted that 5′-RACE is a qualitative detection method that can prove the occurrence of cleavage events, but it cannot quantitatively evaluate the proportion of cleaved mRNA in the total CjAP2 transcript.

To test the functional effects of sequence variants in the putative miR172 target site, the wild-type target site or variant sequences *CjAP2-1-mut5* and *CjAP2-1-mut9* were cloned into a luciferase reporter construct and co-expressed with the miR172 genes in a *Nicotiana* leaf transformation system (Figure 6B). Repression via the target site by miR172 was observed as a reduction in luciferase activity. Inactivation of the target site in the variants was indicated by the recovery of luciferase activity in cells co-expressing the miR172 gene. All four miR172 members were tested. Compared with the wild-type construct, both mutants exhibited significantly higher luciferase activity when co-expressed with miR172-B, -C, and -D, indicating reduced miR172-mediated repression (Figure 6C). The only exception was miR172-A, which did not significantly repress *CjAP2-1*-*mut9* relative to the wild type (Figure 6C). These results suggest that the two mutations differentially affect miRNA binding efficiency, thereby modulating the susceptibility of *CjAP2-1* to post-transcriptional regulation by specific miR172 isoforms.

### 2.6. Functional Analysis of CjAP2 Genes in Arabidopsis

**Figure 7:**
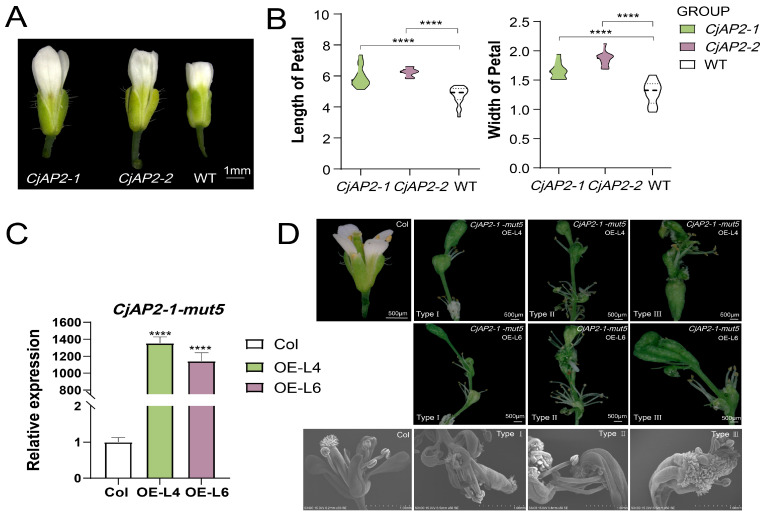
Functional characterization of *CjAP2* and miR172-target mutant in regulating floral development and meristem determinacy. (**A**) Morphological comparison between wild-type *Arabidopsis* and transgenic lines overexpressing *CjAP2-1* and *CjAP2-2*. (**B**) Quantitative analysis of petal length and width in wild-type and transgenic plants. Data represent mean ± SD (*n* = 15). Statistical significance was determined using Student’s *t*-test (**** *p* < 0.0001). (**C**) qRT-PCR analysis of *CjAP2-1-mut5* expression in flower buds of *CjAP2-1-mut5-L4* and *CjAP2-1-mut5-L6* lines. Data represent mean ± SD (*n* = 3); significance was assessed using Student’s *t*-test (**** *p* < 0.0001). (**D**) Representative floral phenotypes of wild-type, overexpression of *CjAP2-1-mut5-L4,* and *CjAP2-1-mut5-L6 Arabidopsis* plants.

To investigate the regulatory roles of *CjAP2* genes in floral development, we generated transgenic *Arabidopsis* lines heterologously overexpressing three constructs: *CjAP2-1*, *CjAP2-2,* and *CjAP2-1*-*mut5* (Supplementary Figure S1). Transgenic lines at comparable developmental stages were selected for phenotypic analysis to assess gene function. We found that overexpression of *CjAP2-1* or *CjAP2-2* resulted in subtle alterations in petal shape with a significant increase in both petal length and width compared to wild-type plants (Figure 7A,B). However, neither gene affected floral organ number or induced homeotic transformations. qRT-PCR analyses confirmed high expression levels of *CjAP2-1*-*mut5* in lines exhibiting strong floral phenotypes (Figure 7C,D). *CjAP2-1*-*mut5* transgenic lines exhibited pronounced floral abnormalities, including ectopic reproductive organ formation, loss of floral determinacy, and fusion of floral organs (Figure 7D). These phenotypes were classified into three categories: (I) loss of floral determinacy, characterized by indeterminate carpel development and repeated carpel initiation; (II) increased stamen number accompanied by partial carpel fusion; and (III) enhanced carpel fusion without a significant change in stamen number.

### 2.7. Disruption of the miR172-CjAP2 Regulation Alters the Expression of Floral Meristem Regulator

**Figure 8:**
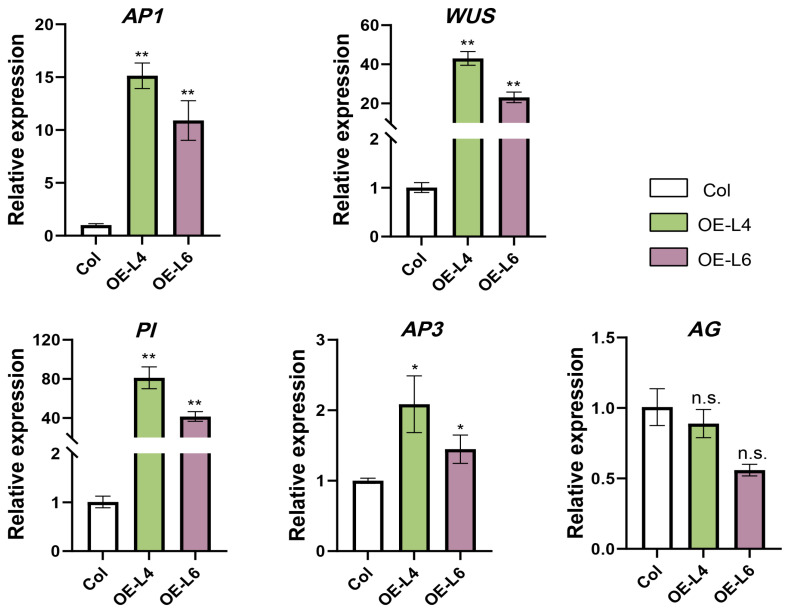
Expression analysis of floral regulatory genes by qRT-PCR. Relative expression levels of selected genes involved in floral development were determined by qRT-PCR. Data represent mean ± SD (*n* = 3). Statistical significance was evaluated using Student’s *t*-test (ns, not significant; * *p* < 0.05; ** *p* < 0.001).

To characterize the molecular basis of the aberrant floral phenotypes observed in *CjAP2-1-mut5* transgenic lines, we performed qRT-PCR analysis of key floral regulatory genes. Compared with wild-type plants, multiple floral identity and meristem determinacy genes were significantly upregulated in *CjAP2-1-mut5* lines, except the *AG* gene (Figure 8). These upregulated genes included the floral organ identity genes *AP1*, *PI* and *AP3*, as well as the meristem determinacy gene *WUSCHEL (WUS*)—all of which have been previously implicated in the genetic network downstream of the miR172–*AP2* module.

These findings support the conclusion that disruption of the miR172–*AP2* regulatory module perturbs floral development. Moreover, they suggest that CjAP2 functions alone with core floral regulators, modulating both floral meristem activity and organ identity specification.

## 3. Discussion

### 3.1. Dosage Effects of miR172–AP2 Regulation Contribute to the Diversity of Floral Forms in C. japonica

Genetic studies in diverse species have established that the miR172–*AP2* regulation is critical for flower development in ornaments. In *Rosa chinensis*, a transposable element insertion in intron 8 of the TOE-type AP2 gene *RcAP2L* produces a truncated transcript resistant to miR172-mediated cleavage. This leads to elevated RCAP2L protein levels, suppression of *RcAG* expression, and consequent conversion of stamens and carpels into petaloid organs [40]. Similarly, in peach, dominant double flowering maps to the Di2 locus, where a deletion removes the miR172-binding site in the euAP2 gene *PETALOSA* (*PET*) (Prupe.6G242400), resulting in ectopic *AP2* accumulation and supernumerary petals [39]. Conversely, recessive double flowering arises from loss-of-function alleles in miR172d, including large deletions and transposon insertions that abolish mature miR172d production, thereby derepressing multiple *AP2* targets [41]. In mei, a 49 bp deletion spanning the miR172 target site in *PmTOE*, coupled with SNPs in the *PmPET* target region, co-segregates tightly with floral doubleness [42], further highlighting the functional centrality of this post-transcriptional regulatory node.

In *C. japonica*, we identified a complex regulatory network comprising four functional miR172 family members and multiple AP2/TOE-like target genes, consistent with lineage-specific expansion observed in other long-lived perennials (Figure 1 and Figure 4). This genetic redundancy likely contributes to robustness in floral patterning but also provides substrate for subfunctionalization and neofunctionalization. Bulked amplicon sequencing of 38 cultivated accessions representing five distinct floral morphologies—from single to fully double forms—revealed extensive nucleotide variation within the miR172-binding domains of *CjAP2*-like transcripts. We found that sequence variations in the miR172 target site regions of the *CjAP2-1* and *CjAP2-2* genes did not introduce any premature stop codons; however, several variants resulted in amino acid substitutions (Figure 5C,D). The functional consequences of these amino acid changes, independent of miR172 regulation, warrant further investigation. Curiously, the allele frequencies of sequence variations in miR172 target sites in different floral groups ranged from less than 0.1% to nearly 100% (Figure 5C,D). Such a low allele frequency cannot be simply explained by heterozygous or homozygous variation at the individual level, which leads to an interesting biological possibility—a genetic chimera. If this somatic mutation occurs in the early stage of flower meristem development, the mutant alleles may be present only in some cell lineages, resulting in an allele frequency much lower than the theoretical genetic value for the entire tissue DNA sample. In this study, the leaf tissue we used for DNA extraction is itself a mixed sample of a cell population, so the extremely low-frequency variation may reflect the phenomenon of somatic mosaicism in individual plants. Notably, several cultivars exhibiting excessive petals carried single- or dinucleotide substitutions in the miR172 complementary sites of *CjAP2-1* (Figure 6). Functional validation via 5′ RACE confirmed that wild-type *CjAP2-1* transcripts are cleaved precisely at the expected position downstream of the miR172-binding site—a mechanism conserved from *Arabidopsis* [34,35,36,37]. We found that two specific dinucleotide variants significantly impaired miR172-directed cleavage efficiency across multiple miR172 isoforms (Figure 5), leading to transcript stabilization and prolonged *CjAP2-1* protein activity during late floral stages. To assess the developmental impact of stabilized AP2 activity, we overexpressed the miR172-resistant *CjAP2-1-mut5* allele in *Arabidopsis*. Transgenic lines exhibited loss of floral meristem determinacy, ectopic reproductive organs, and organ fusion (Figure 7).

Collectively, we demonstrated that variation in floral form diversity in *C. japonica* is regulated by dosage-sensitive perturbations in the conserved miR172–*AP2*-like regulatory module. We revealed that allelic changes on miR172 binding sites of *AP2*-like genes are involved in shaping floral organ identity and meristem determinacy. The repeated, independent recruitment of mutations disrupting miR172–target interactions across distantly related families—including Rosaceae, Solanaceae, and now Theaceae—underscores the evolutionary plasticity and developmental vulnerability of this pathway.

### 3.2. miR172-AP2 Module Regulates Floral Patterning Through Floral Meristem Activity and Determinacy

The miR172-*AP2* regulatory module is a deeply conserved genetic pathway in angiosperms that governs not only floral organ identity but also the activity and determinacy of the floral meristem, as well as the transition from vegetative to reproductive development [36,50]. In *Arabidopsis*, miR172-mediated repression of *AP2* and related TOE family genes is essential for restricting stem cell proliferation and ensuring timely termination of the floral meristem [38,51]. Functional redundancy among MIR172 family members further fine-tunes the process across developmental stages and tissue contexts [52]. Our study in *C. japonica* revealed that natural variation in miR172 targeting sites of *CjAP2* genes perturbs the regulation of the floral meristem. In particular, the cleavage product of *CjAP2* mRNA was detected at the expected miRNA cleavage site by the 5′-RACE experiment (Figure 6A), which provided direct evidence for miR172-mediated *CjAP2* transcript cleavage. However, it should be emphasized that 5′-RACE is essentially a qualitative experiment that can prove the occurrence of cleavage events but cannot quantitatively evaluate the proportion of cleaved mRNA to the overall *CjAP2* transcript. Therefore, although our data support the existence of the cleavage mechanism, it is not certain that this is the only or main way by which miR172 regulates *CjAP2*. Through heterologous expression in *Arabidopsis*, we demonstrated that a miR172-resistant *CjAP2-1* variant (*CjAP2-1-mut5*) does not increase petal number but instead induces severe defects in floral meristem determinacy—including indeterminate carpels, ectopic reproductive organ formation, and organ fusion (Figure 7).

At the molecular level, we observed significant upregulation of *AP1*, *PI*, *AP3*, and *WUS*, all of which are core components of the floral regulatory network downstream of *AP2* (Figure 8). The results are consistent with previous studies demonstrating that derepression of *AP2* results in loss of floral determinacy, proliferation of reproductive organs, and prolonged meristem maintenance, frequently accompanied by altered expression levels of B-class genes and *WUS* [35,38,51,52]. The elevated transcription level of *WUS* is consistent with previous studies demonstrating that repression of *AP2* by miR172 is required to restrict *WUS* temporal–spatial expression in floral meristems [38,53]. As shown in this study, perturbation of miR172–*CjAP2* regulation may compromise this restriction, resulting in prolonged stem cell activity and excessive cell proliferation in organ development of double flowers. Interestingly, the expression of *AG* was not altered in *CjAP2-1-mut5* lines (Figure 8). This indicates that overexpression of miR172-resistant *AP2* leads to loss of floral determinacy independently of *AG* transcript abundance, which is in line with the conclusions of previous research in *N. benthamiana* and other species [53,54].

Together, our results indicate that natural allelic variation at the microRNA172 binding site in cultivated varieties can remodel conserved developmental pathways to create more complex floral morphologies. This work underscores the potential of the miR172-*AP2* regulatory module as a key target in the evolution and domestication of double-flowered ornamentals.

## 4. Materials and Methods

### 4.1. Plant Materials and Growth Conditions

All *Camellia* materials used in this study were obtained from the Camellia Germplasm Nursery of the Research Institute of Subtropical Forestry, Chinese Academy of Sciences (Fuyang City, Zhejiang Province, China; 119°57′ E, 30°04′ N). *Arabidopsis* ecotype Columbia-0 (Col-0) was used for genetic transformation and functional analyses. *Arabidopsis* plants were transformed using the floral dip method [55]. Transgenic seeds were selected on half-strength Murashige and Skoog (1/2 MS) medium supplemented with 10 mg L^−1^ hygromycin. *Arabidopsis* and *N. benthamiana* plants were cultivated in a controlled greenhouse under long-day conditions (16 h light/8 h dark) at 22 °C.

### 4.2. Gene Family and Sequence Analysis

To identify AP2 homologous genes in *C. japonica*, we employed a homology-based search strategy [56]. Specifically, the *Arabidopsis* AP2 protein sequence was used as a query to perform a BLASTp search (E-value cutoff 1 × 10^−15^) against the *C. japonica* sequence database (NCBI BioProject accession No. PRJNA901631) [49]. We extracted all the predicted protein-coding sequences from the *C. japonica* reference genome annotation file and constructed a local protein database for subsequent homology search. Preliminary sequence alignments were performed using BioEdit 7.2.5 software. Phylogenetic analysis of AP2-like family members was conducted using the NJ method implemented in MEGA X 10.0.3 software, with default parameters. The resulting phylogenetic tree was visualized and annotated using the Interactive Tree of Life (iTOL) platform (http://itol.embl.de/, accessed on 2 July 2025). Reference sequences were retrieved from The Arabidopsis Information Resource (TAIR; https://www.arabidopsis.org, accessed on 21 October 2024) and the National Center for Biotechnology Information (NCBI; https://www.ncbi.nlm.nih.gov/, accessed on 21 October 2024). Image processing and figure assembly were performed using Adobe Photoshop CC 2017 and Adobe Illustrator 2024.

### 4.3. Vector Construction and Subcellular Localization Analysis

Full-length coding sequences (CDSs) of *CjAP2* genes lacking stop codons were amplified and cloned downstream of the Cauliflower mosaic virus (CaMV) 35S promoter to generate *CjAP2–EGFP* fusion constructs as described [57,58]. The recombinant plasmids were introduced into *Agrobacterium tumefaciens* strain GV3101 (Weidi, Shanghai, China) and transiently expressed in *N. benthamiana* leaves via agroinfiltration. Following infiltration, plants were incubated in darkness for 3 days. Fluorescence signals were observed using a Zeiss LSM900 confocal laser scanning microscope (Zeiss, Oberkochen, Germany).

### 4.4. qRT-PCR Analysis

Total RNA was extracted using the HiPure HP Plant RNA Mini Prep Kit (Cat. No. R4165-02, Magen, Guangzhou, China) according to the manufacturer’s instructions. First-strand cDNA synthesis was performed using the PrimeScript™ RT reagent kit (Cat. No. RR047A, Takara, Beijing, China). qRT-PCR was conducted on an ABI QuantStudio™ 7 Flex Real-Time PCR System (Thermo Fisher Scientific, Waltham, MA, USA). Relative gene expression levels were calculated using the 2^−ΔΔCT method [59]. *ACTIN* and *GLYCERALDEHYDE 3-PHOSPHATE DEHYDROGENASE* (*GAPDH*) were used as internal reference genes for *Arabidopsis* and *C. japonica*, respectively. Primers for miRNA expression analysis were designed based on the precursor sequences of the four miR172 family members. The precursor sequences were verified using sequence-specific primers and subsequently targeted for quantitative PCR analysis. All experiments were performed with three technical replicates and two biological replicates per sample. Primer sequences are listed in Supplementary Table S1.

### 4.5. Amplicon Sequencing Analysis

Young leaves from different *Camellia* cultivars were collected and classified into five groups according to flower type: single-flowered (*n* = 5), semi-double-flowered (*n* = 9), anemone-flowered (*n* = 7), double-flowered (*n* = 10), and peony-flowered (*n* = 7). Detailed sample information is provided in Supplementary Table S2. For each group, genomic DNA was extracted from the young leaves of each individual, and then the DNA of all individuals in the same floral group was mixed in equal quantities to construct a mixed sample for amplicon sequencing. Full-length genomic sequences of *CjAP2-1* and *CjAP2-2*, including introns, were obtained from the reference genome. The reference genome sequence of *C. japonica* used in this study is from the study of Hu et al. [49]. The genome assembly and annotation data are available from [NCBI Bioproject accession No. PRJNA901631]. Universal primer adapters flanking the predicted miR172 target sites were designed using Primer3Plus (Supplementary Table S1). PCR products of expected size were purified and subjected to high-throughput tracking of mutations (Hi-TOM 1.0) amplicon sequencing for mutation detection [60]. Hi-TOM is an online analysis tool specifically for mutation detection and quantification of high-throughput amplicon sequencing data [60]. Through specific barcode labeling and data analysis processes, the platform can accurately identify low-frequency variations from amplicon sequencing data of mixed samples and quantify the relative abundance of each variation type. The brief process is as follows: PCR amplification of the target region → product mixing library construction → high-throughput sequencing → uploading data to the Hi-TOM platform → platform automatic denoising, alignment and mutation identification → output of each mutation site and its frequency in the mixed sample.

### 4.6. 5′ Rapid Amplification of cDNA Ends (RACE) Analysis

5′ RACE was performed using carpels from the *Camellia* cultivar ‘Naidong’ with the FirstChoice^®^ RNA Ligase-Mediated (RLM)-RACE Kit (Cat. No. AM1700M, Thermo Fisher Scientific, Waltham, MA, USA), with the modification of TAP to obtain 5′ ends. PCR amplification was carried out using PrimeSTAR^®^ Max DNA Polymerase (Cat. No. R045A, Takara, Beijing, China). Amplified fragments were cloned into the pMD™19-T vector (Cat. No. 6019, Takara, Beijing, China) and transformed into *Escherichia coli* DH5α competent cells (Weidi, Shanghai, China) for sequencing validation.

### 4.7. Vector Construction and Dual-Luciferase Assay

The dual-luciferase assay employed the pGreen_GUS_competitor and pGreen_dualluc_3′UTR_sensor vectors. The predicted miR172 target sequence of *CjAP2* and two mutant target sequences were individually cloned into the 3′ untranslated region (UTR) downstream of the firefly luciferase coding sequence in the pGreen_dualluc_3′UTR_sensor vector using double-enzyme digestion and ligation. Meanwhile, the four pri-miR172 sequences were independently cloned into the pGreen_GUS_competitor vector. Recombinant plasmids were co-infiltrated into *N. benthamiana* leaves using *Agrobacterium* (GV3101, OD600 = 0.5)-mediated transient expression. Luciferase activity was measured to assess miRNA-mediated degradation of *CjAP2* transcripts [61]. The system uses two luciferases: firefly luciferase and renilla luciferase. Firefly luciferase is a reporter gene, and its activity is affected by the test sequence (wild-type or mutant miR172 target site) and miR172 co-expression. Renilla luciferase, as an internal reference gene, was constitutively expressed in the co-transfection system to correct system errors such as conversion efficiency, cell number and lysis efficiency between different samples. The normalized relative luciferase activity was obtained by calculating the ratio of firefly luciferase activity to renilla luciferase activity (Firefly/Renilla), which accurately reflected the response of the test sequence to the regulation of miR172.

### 4.8. Image Acquisition and Data Analysis in Arabidopsis

Images of *Arabidopsis* plants were captured at the Forest Insect Laboratory (Room 306) using a VHX-5000 digital microscope (Keyence, Osaka, Japan) and at the Floral Molecular Heritage Laboratory (Room 712) using a stereomicroscope at the Research Institute of Subtropical Forestry, Chinese Academy of Sciences. Morphological measurements were performed using ImageJ2 software. Statistical analyses were conducted using GraphPad Prism 8.

### 4.9. Scanning Electron Microscopy (SEM)

Flowers from transgenic *Arabidopsis* plants were fixed in FAA solution (50% ethanol, 5% acetic acid, and 10% formaldehyde), followed by dehydration through a graded ethanol series. Samples were examined using a Hitachi S-3400N scanning electron microscope (Hitachi, Tokyo, Japan) at the Third Experimental Station of the China National Rice Research Institute (Fuyang District, Hangzhou, China).

## 5. Conclusions

This study demonstrates that the conserved miR172–*AP2* regulatory module plays a pivotal role in the formation of double flowers in *C. japonica*. We identified two AP2-like transcription factors, *CjAP2-1* and *CjAP2-2*, which harbor conserved miR172-binding sites and are broadly expressed in floral tissues, indicating their involvement in flower development. Our results show that miR172 directly cleaves *CjAP2* transcripts and that natural sequence variations at the miR172 target sites can weaken this post-transcriptional repression. Notably, two dinucleotide mutations in *CjAP2-1* significantly reduced miR172-mediated regulation. Functional assays in *Arabidopsis* revealed that expression of a miR172-resistant *CjAP2-1* variant leads to severe defects in floral determinacy and organ identity, accompanied by misregulation of key floral regulatory genes. Together, these findings provide molecular evidence that disruption of the miR172–*CjAP2* interaction is a major driver of double-flower formation in *C. japonica*, extending the conserved role of this regulatory module to woody ornamental plants and offering valuable targets for ornamental breeding.

## Data Availability

The original contributions presented in this study are included in the article/Supplementary Materials. Further inquiries can be directed to the corresponding authors.

## Supplementary Materials

The following supporting information can be downloaded at https://www.mdpi.com/article/10.3390/ijms27062769/s1.

## Abbreviations

The following abbreviations are used in this manuscript:

AFB	AUXIN SIGNALING F-BOX
AG	AGAMOUS
AP	APETALA
ARF	AUXIN RESPONSE FACTOR
CA	Carpel
CaMV	Cauliflower mosaic virus
CDSs	Coding sequences
CUC	CUP-SHAPED COTYLEDON
DEF	DEFICIENS
ERF	Ethylene-responsive factor
GAPDH	GLYCERALDEHYDE 3-PHOSPHATE DEHYDROGENASE
GRF	GROWTH-REGULATING FACTOR
Hi-TOM	High-throughput tracking of mutations
iTOL	Interactive Tree of Life
miRNAs	microRNAs
MS	Murashige and Skoog
MYB	V-MYB AVIAN MYELOBLASTOSIS VIRAL ONCOGENE HOMOLOG
NAM	NO APICAL MERISTEM
NCBI	National Center for Biotechnology Information
NF-YA	NUCLEAR TRANSCRIPTION FACTOR Y SUBUNIT ALPHA
NJ	Neighbor-joining
PHB	PHABULOSA
PI	PISTILLATA
PE	Petal
PET	PETALOSA
qRT-PCR	Quantitative real-time PCR
RACE	Rapid amplification of cDNA ends
RLM	RNA ligase-mediated
SEP	SEPALLATA
SE	Sepal
SEM	Scanning electron microscopy
SHP	SHATTERPROOF
SMZ	SCHLAFMÜTZE
SNZ	SCHNARCHZAPFEN
SNPs	Single-nucleotide polymorphisms
SPL	SQUAMOSA PROMOTER-BINDING PROTEIN-LIKE
STA	Stamen
STK	SEEDSTICK
TAIR	The Arabidopsis Information Resource
TCP	TEOSINTE BRANCHED1-CYCLOIDEA-PROLIFERATING CELL FACTOR
TIR	TRANSPORT INHIBITOR RESPONSE
TOE	TARGET OF EAT
UTR	Untranslated region
WUS	WUSCHEL
