# Supplementary figures and images for: miR172-Mediated Repression of APETALA2-like Genes Regulates Floral Meristem Activity During Double-Flower Formation in Camellia japonica
Source: Int J Mol Sci. 2026 Mar 18;27(6):2769. doi: 10.3390/ijms27062769 (PMC13027160; doi:10.3390/ijms27062769)

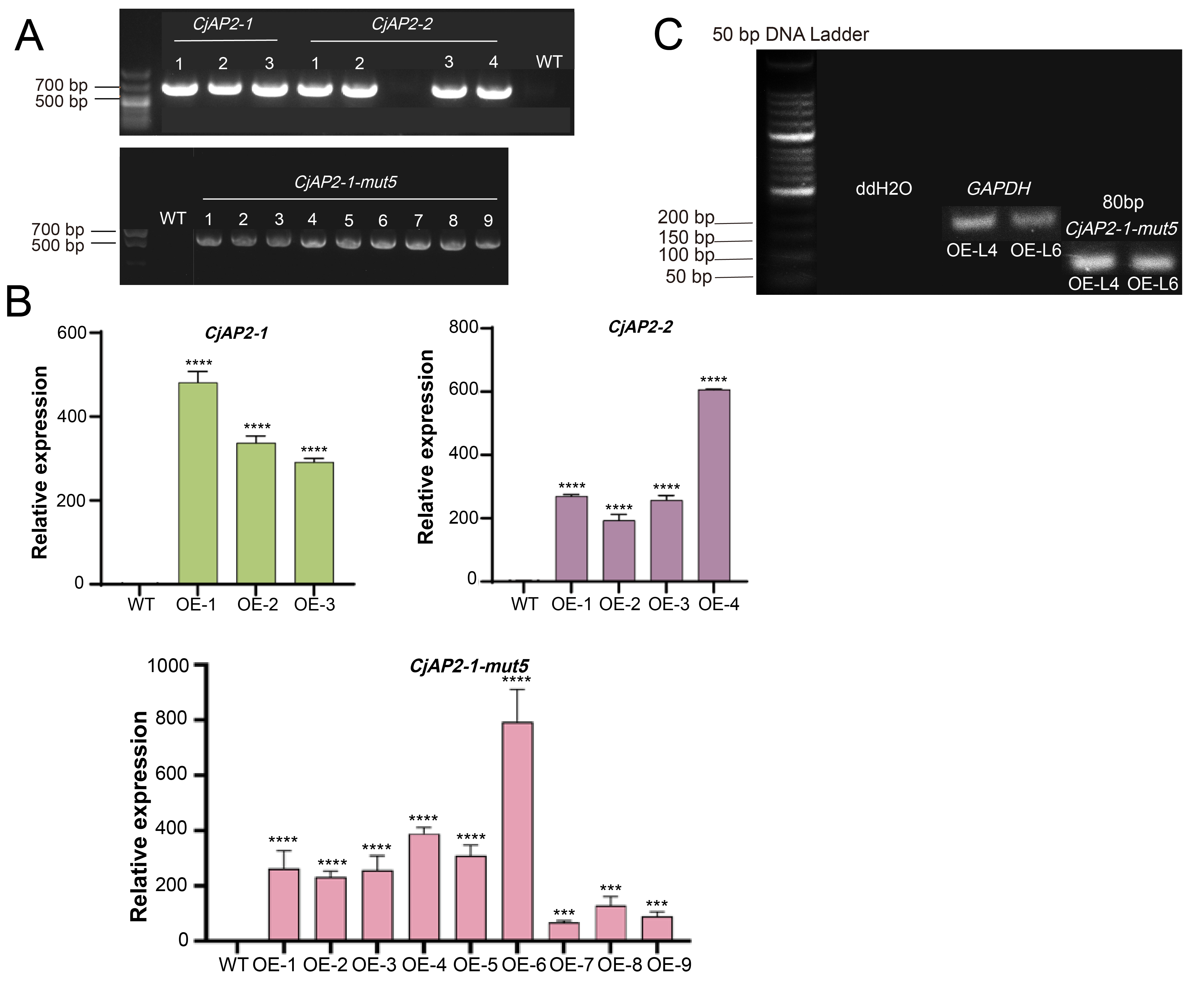

Supplement: Supplementary file 1 [file ijms-27-02769-s001.zip › Figure S1.png]
